# Exploring implementation processes in general practice in a feedback intervention aiming to reduce potentially inappropriate prescribing: a qualitative study among general practitioners

**DOI:** 10.1186/s43058-020-00106-5

**Published:** 2021-01-07

**Authors:** Kirsten Høj, Anna Mygind, Flemming Bro

**Affiliations:** 1 Research Unit for General Practice, Bartholins Allé 2, 8000 Aarhus C, Denmark; 2 Department of Public Health, Aarhus University, Aarhus, Denmark

**Keywords:** Potentially inappropriate prescribing, Feedback, Intervention, Quality improvement, Primary care, General practice, Normalisation Process Theory

## Abstract

**Background:**

Potentially inappropriate prescribing (PIP) has been linked with adverse health outcomes and increased healthcare costs. Feedback interventions targeting PIP have shown promising results. However, translation from research to everyday practice remains a challenge. With the Normalisation Process Theory (NPT) as overarching framework, we aimed to explore the implementation processes performed by general practices in a real-life, quality improvement intervention using feedback on practice-level prescribing.

**Methods:**

All 376 general practices in the Central Denmark Region received a prescribing feedback intervention targeting selected types of PIP. Six months later, they received an evaluation questionnaire, to which 45% responded. Among 102 practices reporting to have made changes in response to the intervention, we conducted individual, semi-structured interviews with ten GPs. Maximum variation was sought in terms of baseline prescribing status, implementation activities, practice type and geographical location. The interviews were analysed thematically using NPT.

**Results:**

The implementation processes in general practice reflected the four NPT constructs. Key motivators for implementation included the GPs’ professional values and interests, but pragmatic considerations were also of importance (*coherence*). A collective versus an individual approach to the engagement and planning of the implementation process (*cognitive participation*) was observed. Similarly, a distinction was evident between practice-level actions involving the entire practice team as opposed to individual-level actions performed by the individual GP (*collective action*). Several challenges to the implementation processes were identified, including patient influences and competing priorities at multiple levels (*reflexive monitoring*). Additionally, internal evaluation and normalisation of new practices occurred in varying degrees.

**Conclusion:**

NPT provided a useful framework for understanding implementation processes in general practice. Our results emphasise that clear professional aims and feasible content of interventions are key for GP motivation. This may be ensured through cooperation with GPs’ professional organisation, which may strengthen intervention legitimacy and uptake. Two main implementation strategies were identified: practice-level and GP-level strategies. Intervention developers need to recognise both strategies to deliver intervention content and implementation support that promote sustainable improvements in prescribing practice. Competing demands and patient influences remain important challenges that need to be addressed in future studies to further facilitate the reduction of PIPs.

**Supplementary Information:**

The online version contains supplementary material available at 10.1186/s43058-020-00106-5.

Contributions to the literature

- Interventions aiming to change the prescribing behaviour in general practice have had varying success, and, despite promising results from feedback interventions, translating research into clinical practice remains a challenge.
- This paper provides important insights into the mechanisms of real-life implementation processes in general practice, including facilitation through professional values and pragmatic considerations, action through practice-level or individual-level strategies, and the challenges of competing priorities and patient influences.
- This knowledge can help intervention planners develop feasible interventions and implementation support to general practices to promote sustainable changes in clinical prescribing practice.

## Background

Potentially inappropriate prescribing (PIP) in primary care is associated with adverse health outcomes and increased healthcare utilisation [1–3]. PIP occurs when the actual or potential harms of treatment outweigh the expected benefits and is observed in on third of primary care patients above the age of 65 years [4, 5]. Further, large practice variation has been identified in the quality and safety of prescribing [6, 7]. However, efforts to change clinical practice in primary care have shown varying success so far [8].

Among specific quality improvement (QI) interventions in primary care, audit and feedback and continuing education have been cited as having the greatest effect on patient outcomes [9]. Audit and feedback provides health professionals with quantitative summaries of their clinical performance [10]. This strategy has become increasingly popular, and various terms have been used. In this paper, we use the simple term *feedback*.

Feedback is typically given for a specified time period in which the recipient’s performance is compared against the performance of peers or the recipient’s own performance over time [11]. Feedback is thought to work by raising the recipient’s awareness of a suboptimal performance, thereby prompting action to improve the performance [12]. A recent Cochrane review, including 140 randomised trials conducted over a range of healthcare settings and targeted behaviours, concluded that feedback generally leads to small, but potentially important improvements in professional performance [13]. Noteworthy, the review showed a considerable effect among studies specifically targeting prescribing. Consistently with these findings, a Scottish cluster-randomised trial in primary care showed that feedback on practice-level prescribing could help reduce high-risk prescribing [14].

In contrast, Trietsch et al. recently found that the positive results obtained in earlier, well-controlled feedback studies on prescribing were not confirmed when the feedback strategy was introduced at large scale in real-life primary care [15]. Similarly, Wagner et al. showed that the implementation of real-world feedback interventions did not lead to the desired QI activities [16]. Thus, despite promising results from feedback interventions in primary care, it remains a challenge to translate research into clinical practice. Evidence suggests that the feedback effectiveness depends on the baseline performance level, feedback method used, health professional receiving feedback and the context in which feedback takes place [10, 13]. However, little is known about how general practitioners (GPs) take action to change their prescribing habits when receiving prescribing feedback [12].

The Normalisation Process Theory (NPT) is a conceptual model that offers an explanatory framework for understanding the mechanisms of implementing interventions [17]. The NPT comprises four main constructs affecting routine embedding: *coherence, cognitive participation, collective action* and *reflexive monitoring*. Coherence refers to the participants’ individual and collective understanding of the intervention. Cognitive participation relates to the engagement in and planning of the implementation work. Collective action refers to the operational work done by participants to implement the intervention. Finally, reflexive monitoring focuses on how participants assess their progress and understand how the intervention affects themselves and others around them. An NPT-led review recently concluded that effective interventions tend to act across several NPT constructs, in particular through collective action and reflexive monitoring, while less effective interventions tend to focus on coherence or the early stages of cognitive participation alone [18].

With NPT as our guiding framework, we aimed to explore implementation processes performed by general practices in a real-life QI intervention using feedback on practice-level prescribing.

## Methods

### Design and setting

This qualitative study involved semi-structured interviews with GPs participating in a QI project focusing on selected PIPs (*Medicin i Midt*). The QI project was conducted in 2017–2020 in a cooperation between the Central Denmark Region and the regional Organisation of General Practitioners (OGP). This paper conforms to the Standards for Reporting Qualitative Research (Additional file 1) [19].

### Intervention: information packages

The intervention consisted of eight information packages each targeting selected types of PIP. These were distributed by surface mail in two rounds to all general practices (*n* = 376) in the region. The first four packages, delivered in September 2017, targeted dipyridamole, proton-pump inhibitors (PPIs), opioid patches and medications for overactive bladder-syndrome. In February 2018, four additional packages targeting non-steroidal anti-inflammatory drugs, antidepressants, oral opioids and inhalation steroids were distributed.

Each information package included four components: (1) statistics on practice-level prescribing (including the defined daily dose (DDD) and number of patients receiving the specific drugs) compared to other practices in the region, (2) recommendations about rational prescribing for the targeted PIP, (3) instructions on how to identify relevant patients in the electronic medical record (EMR) system and (4) inspiration for an action plan to implement the information packages. Implementation of the intervention was voluntary, no fees were provided and practices were free to organise the implementation as they saw fit. All practices received updated prescribing feedback on the targeted PIPs (component 1) by surface mail every sixth months during the project period (2017–2020).

### Implementation context

In Denmark, general practices are mainly independent, physician-owned small businesses that provide primary medical care under a collective agreement with the Danish Regions [20]. There are no patient-paid user fees for primary care services. The services are tax-financed and administered by the Danish Regions, including reimbursement of expenses for drugs eligible for subsidy.

### Sampling strategy

**Fig. 1:**
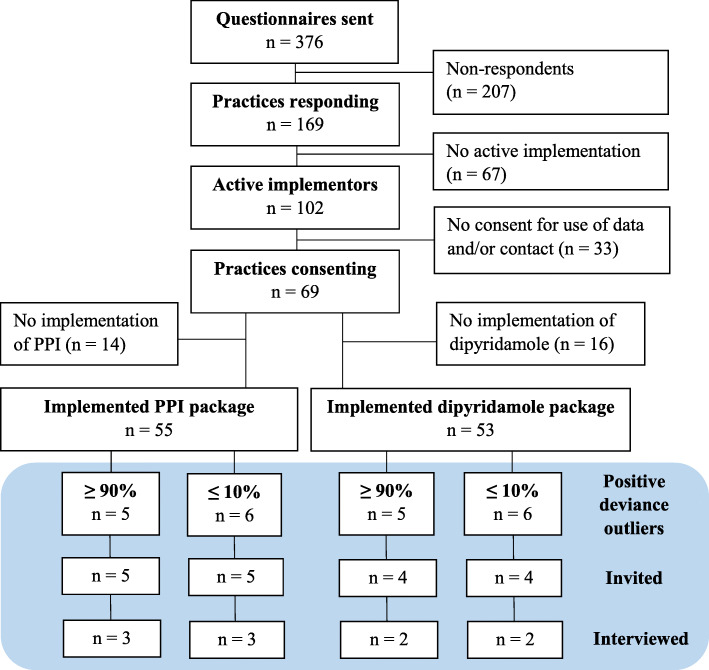
Flowchart of the recruitment of GPs for the interviews. GP, general practitioner; PPI, proton-pump inhibitor

As part of the QI project evaluation, a questionnaire based on NPT concerning the first four information packages was sent by surface mail to all practices in February 2018. The questionnaire data served as basis for a strategic sampling of informants for this qualitative study. Of 376 practices, 169 returned the questionnaire (Fig. 1). Among the respondents, 102 practices reported to have actively implemented the information packages (i.e. taken action to change the prescribing of specific drugs). Of these practices, 69 consented to further contact and data use for research. Eligible informants were selected through a positive deviance approach to ensure variation in baseline prescribing levels [21]. We used baseline prescription data for dipyridamole and PPI acquired from the Danish National Prescription Registry [22] to identify the high-end (≥ 90th percentile) and low-end (≤ 10th percentile) prescribers. Of the 69 active practices, 53 reported to have worked with the package concerning dipyridamole and 55 with the package concerning PPI. Among these, the positive deviance approach yielded in total 22 practices. Potential informants were further selected by criterion sampling to ensure variation in practice type and geographical location. This process yielded 18 practices, which received an invitation by surface mail asking one GP from each practice to participate in an individual, semi-structured interview. In total, ten GPs accepted the invitation.

### Data collection

All interviews were conducted by KH between 23 May 2018 and 3 July 2018. The interview guide was based on NPT [17]. Further, as patient influence was a strong presence in the first interview, this theme was added to the interview guide. The 1-h interviews were conducted at a time of the participants’ choosing at their clinic. Field notes were written immediately after each interview to enable preliminary analysis. The interviews were audio-recorded and transcribed verbatim.

### Data analysis

All transcripts and field notes were read and coded independently by KH. Key data units were coded through an iterative process, in which the coding list was continuously developed. Furthermore, for each interview, a drawing was made to visualise the processes and the persons involved in each step. When the coding process was complete, codes with shared features were grouped into emergent themes that were finally assembled into the four overarching NPT constructs. In addition, the drawings were merged to provide an overview of similarities and differences across practices. Thus, data were analysed both inductively and deductively in the process. All authors engaged in ongoing discussions about and agreed upon the emergent themes.

## Results

The informants varied with respect to baseline prescribing level, gender, years of experience as GP, practice type and geographical location (Table 1). The data analysis exposed several emergent themes that came into play during the implementation process. In the following, the themes will be presented according to the four overarching NPT constructs.

**Table 1:** Characteristics of the interviewed GPs (*n* = 10)

ID	Gender	Years of experience as GP	Practice type	Practice-level prescribing status
GP1	Female	≥ 20	Single-handed	High-end
GP2	Female	≥ 20	Single-handed	High-end
GP3	Female	10 to 20	Single-handed	Low-end
GP4	Male	10 to 20	Partnership	Low-end
GP5	Male	≤ 10	Partnership	Low-end
GP6	Male	10 to 20	Partnership	Low-end
GP7	Female	10 to 20	Partnership	High-end
GP8	Male	≥ 20	Single-handed	High-end
GP9	Female	≥ 20	Single-handed	Low-end
GP10	Female	≥ 20	Partnership	High-end

*GP* general practitioner

### Coherence

Coherence concerns how the participants understand the intervention. In this initial phase of the implementation, the GPs articulated different reflections upon receiving the information packages.

#### Professional values and pragmatic considerations

All the GPs found that management of PIPs was an important task in general practice, and professional values were the key drivers for working with the information packages. One GP explained,

You want to do what is best for the patient; you also want to comply with the clinical standards. (GP1)

Some GPs saw the information packages as a good opportunity to be updated and inspired to focus on specific themes to improve prescribing quality. One GP said,

[The content of the information packages] was focused and well articulated. I think it has been a great initiative […]. And something you wanted to engage in. (GP8)

Some GPs were motivated by the feedback as it gave them insight and opportunity to reflect on own prescribing behaviour. Especially, some GPs in single-handed practices found it valuable because they usually had no one to discuss with in everyday clinical practice. One GP explained,

I […] believe that this [feedback] is helpful for me. Because I sit here as a single-handed GP, trying to do the best possible for my patients. But I am also aware that you are more likely to step into a pitfall if you do exactly the same thing every time you encounter certain problems. (GP3)

Other GPs praised the information packages for being manageable, although some packages were considered more likely to succeed than others, e.g. due to a limited number of patients or a relatively simple clinical issue. One GP said,

[The packages concerning dipyridamole and betmiga] were easy to get started with. And there was also a chance for a quick gain and a more clear gain. Whereas […] there were much more profound issues for pain patients. (GP4)

Thus, the GP’s decision whether to work with the information packages was also influenced by pragmatic considerations regarding the required work efforts, including the number of patients and the complexity of the clinical issue.

#### Clinical autonomy and professional interest

In contrast to the enthusiastic reception of the information packages among some GPs, other GPs initially took a more critical approach. They perceived the purpose of the intervention as a control or cost reduction tool initiated by the regional authorities and regarded the intervention as an interference with their clinical autonomy. One GP said,

At first, you may take a slightly negative approach and think ‘Oh, they [the region] always interfere and control’ […] to make us change behaviour for purely financial reasons, and not what is best for the patients. And [I feel] a little annoyed that there is yet another thing that we must consider. (GP3)

The peer-comparison of prescribing levels also got a tepid reception by some GPs, who emphasised that differences in prescribing behaviour could be attributed to different patient populations, e.g. older and sicker populations, rather than differences in clinical behaviour. However, after having further studied the information packages, the critical GPs had generally taken a more positive view. One GP explained,

Once you have put away the negative [attitude] and said ‘okay’, this can actually be seen as an opportunity to check if things are done properly, and if you have some bad habits. (GP3)

Some of the GPs were also clearly aware that the OGP supported the intervention. This was important for their choice to work with the packages. One GP said,

What [the OGP] supports, I can also support […] because I like us to appear as an organisation or a unity. General practitioners, we stick together, and when we decide something, we also do something about it. (GP6)

Thus, the intervention was initially perceived by some GPs to interfere with their clinical autonomy. However, particular among some of the critical GPs, professional interest was an important facilitator for implementation. We found no pattern in the GPs’ reflections in terms of baseline prescribing levels.

### Cognitive participation

Cognitive participation involves the engagement in and planning of the implementation work. In this phase, the GPs took either an individual or a collective approach.

#### Individual versus collective approach

**Fig. 2:**
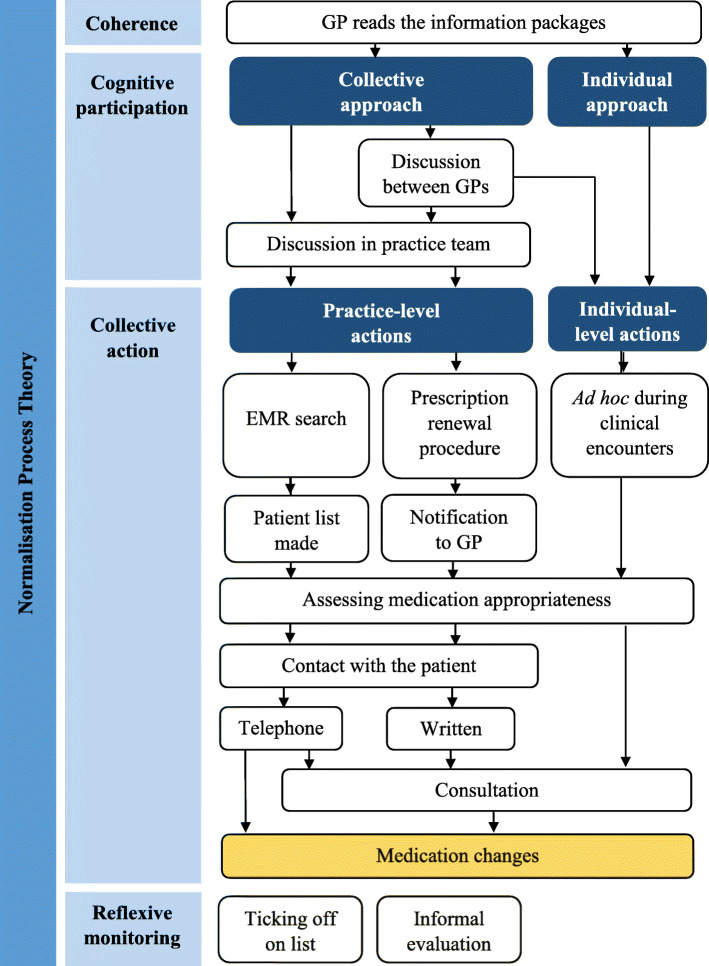
Overview of implementation processes in general practice in response to the prescribing feedback intervention. GP, general practitioner; EMR, electronic medical record; PR, prescription renewal

The information packages were processed by the GPs either individually (i.e. the GP alone) or collectively (i.e. discussed in the GP group or in the entire practice team (GPs and staff)) (Fig. 2). In single-handed practices, the GP decided whether and how to work with the implementation. Some chose to take action on their own, while others involved their practice staff. In partnership practices, the GPs agreed whether or not the practice should work with the information packages and in which way. Some partnership practices appointed one GP with responsibility for coordination.

### Collective action

Collective action refers to the active work that is carried out to implement new practices. In this operational phase, the implementation actions differed both between the individual practices and between the individual information packages. Overall, two levels of implementation actions were identified, namely practice-level and individual-level actions (Fig. 2).

#### Practice-level actions

Practice-level actions involved the systematic approaches used by the practices to identify patients with selected PIPs. Some practices made a search in the EMR system, e.g. through anatomical therapeutic classification codes or prescriptions, to create a list of patients who were prescribed the medication in question. This activity was separate from the usual workflow and was conducted by a GP or a practice staff member. The list was examined by a GP to assess if the individual patient still got the medication and, if so, whether it would be relevant to consider medication changes. This EMR approach was primarily taken when the number of patients using the medication in question (e.g. dipyridamole) was sufficiently low to be managed this way.

In other practices, a new procedure within the existing workflow was developed to ensure that relevant patients were identified when the opportunity arose upon prescription renewal. In case of a renewal request for selected medications, the staff was instructed, orally or in writing, to notify the GP (e.g. through the GP’s electronic task list). The GP would then assess whether it would be appropriate to consider medication changes in the patient.

In both approaches (EMR and prescription renewal), the patients eligible for medication changes were contacted by telephone or in writing (letter or e-mail). If the issue was considered to be a simple case, some GPs called the patient and discontinued the medication by telephone. For instance, deprescribing of dipyridamole was considered straightforward because this medication is not used for symptom relief. Hence, the GPs did not expect the patients to reject the deprescribing proposal. In other cases, the staff called the patient and scheduled a consultation to discuss relevant medication changes. These consultations were primarily conducted by a GP, but some practices had trained a nurse to discuss medication changes, e.g. deprescribing of PPIs. For written requests, standard information on the background was enclosed, and the patient was generally encouraged to book an appointment. However, one practice encouraged patients to self-discontinue PPI by following a standardised deprescribing plan enclosed in the letter.

#### Individual-level actions

Individual-level actions involved attempts by the individual GP to identify relevant patients during clinical encounters (e.g. annual chronic care consultation or ordinary consultation booked for another reason). This ad hoc approach was perceived by the GPs as increased awareness in daily clinical practice. The GP would discuss medication changes with relevant patients if the opportunity arose or would plan to address the issue at another encounter if the timing was considered inappropriate. For some GPs, the individual-level action was preceded by individual cognitive participation, whereas other GPs had collectively discussed the content of the information packages, including the feedback, but had left the implementation actions up to the individual GP.

In some practices, the individual-level actions were the only implementation strategy taken. In other practices, the GPs used practice-level actions for one or two packages, while the remaining packages were implemented ad hoc by individual-level actions.

### Reflexive monitoring

Reflexive monitoring involves participants’ reflections on the ways in which the intervention affects themselves and others and how they evaluate and value their implementation efforts. The GPs articulated different reflections on the challenges relating to the implementation work, and internal evaluation and normalisation of new practices occurred in varying degree.

#### Competing priorities

At the practice level, the greatest challenge was prioritisation of time to get started, and many GPs struggled to balance the demands on their time. One GP said,

Allowing the time for it. And just to get it started. That is definitely […] the big hurdle. (GP6)

Some GPs also indicated that it was a challenge to reach agreement with the other GPs in the practice on whether or not to take action due to opposing collective priorities or different attitudes to the information packages. One GP explained,

The real challenge was, perhaps, to say; shouldn’t we do this? To the others. Knowing, full well, that they thought they had plenty to do. Then they look at me like, no. […] Where you know that it’s not the professional competency that’s the problem, but it’s the time. (GP4)

In such cases, disagreements had been resolved by granting the co-ordinating responsibility to a highly engaged GP to minimise the workload on less engaged GPs.

At the individual GP level, competing priorities and time pressure during patient encounters were reported as barriers for putting medication changes on the agenda. A further challenge was the dilemma between considerations for the individual patient and for society. Some GPs found that the societal focus was given priority by the intervention; they experienced discrepancy between their ideals of patient-centred care and the goals of the intervention to increase quality. One GP expressed it this way,

Whose expectations must I meet? Well, first of all, my own professional expectations of doing a proper job. But […] what does the patient really want? We say that we are here for the patients. Not for the large group in society. (GP4)

Some GPs found that the overall messages in the information packages did not truly mirror the reality and appealed to the region not to enforce medication changes with cost savings in mind. In contrast, other GPs did not find the population-based approach directly opposing to a patient-centred approach. One GP said,

Each individual does not get the same message. In fact, you set an individual success criterion for each patient. Because it is obviously not the same thing that applies to every patient. In this sense, I think that it is patient-centred. And the region may have some hidden agenda behind this, and I may have another. But the end goal can still be the same, namely that the dose is lowered or the medicine is stopped. (GP3)

#### Patient influences

All the interviewed GPs experienced that patients rejecting proposed medication changes were a challenge for implementing the information packages. One GP explained,

[The patients] are not happy about losing their medication. It is a bit like, ‘No, why can’t I have it?’. So, we try our best. But we don’t get happy patients out of it. (GP1)

Some GPs found this a major problem, specifically among the elderly patients, while other GPs believed that it concerned mostly a small group of so-called difficult patients. Some GPs also stated that rejections were seen mainly for specific types of medications, such as symptom relief (e.g. PPIs) and highly addictive drugs.

In case of disagreement between the GP and the patient on the treatment goals, variation was found in the GPs’ perceptions of the situation. Some GPs felt frustrated that they could not convince the patient of their own agenda (i.e. compliance perspective) and perceived deviance from the official recommendations as compromising to their professional integrity in order to maintain a good doctor-patient relationship. Other GPs stressed the importance of respecting the patient’s autonomy and, to some extent, meeting the patient’s preferences. One GP said,

We must also respect people’s own experiences and wishes, at least part of the way. (GP8)

GPs with this viewpoint (i.e. adherence perspective) sought to elicit the patient’s experiences and preferences to reach a shared decision, and they perceived deviation from recommendations as patient-centred care. Some GPs also specified that the timing was important for successful medication changes. One GP explained,

If you have an unresolved case of early retirement or similar, it may not be the right time to get off medication. The chance of success is zero […]. But when it is over, then you might be able to do it. (GP4)

According to the GPs, rejection could also come from relatives or care home assistants, who were feeling unsafe about deprescribing medication on behalf of the patient. This could often be resolved through dialogue. Some GPs experienced that the care home staff and the district nurses often took a positive approach to a suggestion for deprescribing in elderly patients. Likewise, some GPs experienced that many patients accepted the offer to change their medications when the reasons were thoroughly explained.

#### Evaluation and normalisation

Some GPs followed up on the outcome of their implementation process (e.g. by ticking patients off on the list of eligible patients), while other GPs did not. Particularly, some practices criticised their EMR system for not allowing easy extraction of the desired information. Evaluation of the actual processes occurred informally and only in some practices.

The intervention contributed to making some practices change or adopt new routines in connection with prescription renewals (e.g. of PPIs), whereas the EMR approach was regarded as a one-time effort. Nonetheless, all the interviewed GPs stated that they had generally become more aware of PIPs. One GP stated,

[The information packages] probably make us more aware of how we prescribe medication, I think. On the whole. (GP1)

## Discussion

### Main findings

In this real-life QI project using feedback on practice-level prescribing, we found that the implementation processes used in general practice reflected the four NPT constructs. The GPs’ professional values and interests served as key motivators for ensuring *coherence*, although pragmatic considerations on feasibility were also of importance. *Cognitive participation* was found to occur as either a collective or an individual approach. Similarly, in *collective action*, a distinction was made between practice-level actions involving the entire practice team and individual-level actions performed by the individual GP. In *reflexive monitoring*, a range of challenges that influenced the implementation processes was identified, including patient influences and competing priorities at multiple levels. Additionally, we found that internal evaluation of implementation processes and outcomes as well as normalisation of the new practices occurred in varying degree.

### Comparison with existing literature

Viewing our results through an NPT perspective allowed us to compare our findings with those of other studies, despite differences in intervention design and target behaviour.

#### Coherence

In line with previous research [23–25], our results imply that the key motivator in GPs for implementing feedback interventions is the ambition to do a good job. Our findings further indicate that GPs also consider the expected work effort, including how realistic it is to make the desired changes in daily practice. These findings emphasise that aligning interventions with the GPs’ professional values and clinical context is imperative for successful implementation.

Although GPs generally consider it a professional obligation to engage in prescribing behaviour, it can be perceived as an imposing constraint or a control function when derived externally [26, 27]. This contradiction was also apparent in our study in both high-end and low-end prescribers. A commonly reported reason for this reaction is a perceived threat to the GPs’ clinical autonomy [27–29]. In our study, the support to the intervention from the OGP reduced the opposition, particularly among the critical GPs. This draws attention to the significance of cooperating with the GPs’ professional organisations to enhance the legitimacy of interventions and increase the professional interest among GPs.

#### Cognitive participation

Our findings of a distinction between collective and individual approaches to implementation planning resemble the findings of Grant et al., who investigated how GPs implement research evidence into everyday prescribing processes [30]. They described two different types of prescribing behaviours. Macro-prescribing involves collective policy decisions made at the practice level; it considers evidence in light of the average patient. Micro-prescribing involves decisions made by the individual GP during consultation; it considers the views, preferences and circumstances of a specific patient. Grant and colleagues showed that practices with high prescribing quality used a combination of macro- and micro-prescribing, while a practice with low prescribing quality used only micro-prescribing. Hence, collective discussion and agreement on practice protocols were associated with high prescribing quality. Similarly, a recent Danish study found that practice meetings and protocol development were associated with higher quality of care, although these associations were less evident in single-handed practices compared to partnership practices [31]. Noteworthy, some partnership practices in our study used a co-ordinating GP to plan the implementation process. This may enable implementation in practices where not all GPs are fully engaged, although it might not be possible in case of strong opposition to the intervention [32].

#### Collective action

The pattern of practice-level and individual-level implementation actions reflects the findings of a Danish study exploring how GPs implement clinical guidelines in everyday practice [28]. This suggests that GPs generally operate with these two types of implementation actions, whether this is in response to a new clinical guideline or a feedback intervention.

The individual-level implementation actions were also demonstrated by Ivers et al. in a feedback intervention, where GPs commonly reported that they intended to improve their performance by being more mindful of relevant targets during patient encounters [23]. Although the individual-level approach to implementation is straightforward, it is highly susceptible to competing priorities in everyday practice [23, 24, 33]. Thus, solely relying on the individual GP to identify patients with PIPs is likely to increase the risk of poorer quality and jeopardising the safety of prescribing [30].

In contrast, practice-level actions have the advantage of being systematic across the entire practice population. In our study, the EMR approach provided an immediate overview of all relevant patients, which worked well in some practices. Other GPs experienced technical difficulties with this approach; a problem that has also been reported elsewhere [23, 34]. This implies that the EMR functionalities must be further developed to support the leverage of data, if the electronic system is to become an integral part of QI work in general practice. Another important observation from the EMR approach was that the practices performed this as a separate on-off activity. This may be appropriate, for instance when the goal is to deprescribe obsolete treatments. However, for regularly prescribed medications, future patients with PIPs may not benefit from this approach alone.

The new prescription renewal approach involved a systematic change in the existing workflow to continually identify relevant patients. This may afford a more sustainable change in the prescribing of certain PIPs [30]. A limitation is that it requires an active enquiry by the patient. Thus, the time to reach all relevant patients is extended. Some patients may also have their prescriptions renewed by another physician [35] and may be overlooked. Another consideration is that it might require a more extensive effort to change existing workflows than to conduct a one-off activity. In conclusion, a combination of complementing practice-level actions integrated into the existing workflow seems to be the most optimal way forward to ensure identification and management of both existing and future patients with PIPs.

#### Reflexive monitoring

Research shows that many GPs struggle with integrating QI concepts into their practices [34, 36–38]. Consistent with prior findings [23, 34, 36], our study showed that time constraints at both the organisational and the individual levels were dominating challenges that forced the practices to prioritise their time. A less noticed issue is the tensions that may arise in practices during the implementation process, as practices must make resource trade-offs between feedback efforts, other QI efforts and the daily clinical work [34, 38]. In line with the findings of the Data-driven Quality Improvement in Primary Care (DQIP) trial, [38] our results show that practices can experience challenges in the initial phases of the implementation process (i.e. coherence and cognitive participation) due to different understandings of the intervention or opposing collective priorities. As all practices in our study were active implementers, they found ways to overcome such issues. However, internal differences may represent an important barrier in other practices.

Our results highlight the professional dilemma that arises when the patients’ priorities do not align with the recommendations of the feedback intervention and display different GP responses to this situation. As found in earlier work [23, 34, 39], some GPs in our study perceived discordance between the QI targets and their ideals of patient-centred care. In some cases, this involved meeting the patients’ preferences despite deviating from the official recommendations. In contrast, other GPs perceived deviation from the recommendations as a compromise on their professional integrity made in order to maintain a good doctor-patient relationship. Thus, our results add to the growing literature on the complexities of PIP management in primary care [35, 40] by emphasising patient influences as a challenge, and yet a necessity, for true patient-centred prescribing.

### Implications

Our results illustrate that the NPT provides a useful framework for understanding the different aspects of the implementation process. They further emphasise that QI developers need to consider all constructs when developing interventions. A key insight gained from our study is the vital importance of aligning interventions with the GPs’ professional values, interests and clinical context to enhance coherence and cognitive participation. As suggested by our results, this may be achieved by close cooperation with the GPs and their professional organisation.

The overarching pattern of practice-level and individual-level implementation strategies has important implications for future QI interventions. Consistent with previous research, our findings imply that effective implementation requires interventions with flexible content that allows the practices to tailor the implementation processes to suit their individual practice context and patient population [38, 41]. Furthermore, the intervention support should be flexible to assist different practice types in their implementation efforts. For instance, practices relying on individual-level implementation may benefit from support to facilitate the organisational processes that will underpin practice-level implementation in cognitive participation and collective action [42]. Contrarily, practices that already perform practice-level implementation may benefit from specific support, such as delivery of patient lists or training of practice staff, to further support collective action and reflexive monitoring [10, 43].

Importantly, intervention developers need to consider how to deal with the barriers of competing priorities and patient influences to support GPs in their management of PIPs. While some evidence supports elements such as financial incentives [14], direct-to-consumer information, or decision aid tools for patients [44–46], more research is needed to elucidate these areas.

### Strengths and limitations

A major strength of this study is the embedded design within a real-life QI project. This provides valuable insights into the real-life processes that general practices carry out to implement new habits. Another important strength is our systematic and well-informed sampling of informants; we used a questionnaire survey combined with register-based prescription data. In particular, the positive deviance approach ensured important variation in baseline performance. These sampling characteristics strengthened the external validity of our results.

A limitation is that, in order to explore implementation activities, we included only GPs implementing the intervention as informants. This limited our opportunity to explore the barriers to initiating implementation. In addition, our study reveals only activities, barriers and enablers as they were perceived by the GPs. Interviews with practice staff and observations of everyday practice in the clinics may have revealed more nuanced processes and thereby identified other factors influencing the implementation work. We tried to accommodate this limitation by conducting the interviews in the GPs’ own clinics, thereby making the informants speak from a position close to their everyday work life. Furthermore, the interviewer’s clinical experience from general practice ensured a good understanding of the everyday work-life conditions. Still, the interviewer, being a clinician researcher, could also have introduced clinical bias into data collection and interpretation. However, the use of deductive coding, based on a theoretical framework, and the cross-disciplinary backgrounds of the research team strengthened the validity of the results.

## Conclusion

NPT provided a useful framework for identifying and understanding key aspects that influenced the implementation processes in general practice. Implementation was motivated by the professional values and interests of the GPs, but pragmatic considerations were decisive. Thus, interventions should have a clear professional aim and a feasible, rather than an ideal, message. This may be achieved through cooperation with GPs and their professional organisation, which in addition may enhance the legitimacy and uptake of the intervention. The included practices took action in various ways to change the prescribing practice. Overall, two principal strategies were used: practice-level and GP-level strategies. It is essential to recognise the existence of both approaches to develop targeted intervention content and implementation support that may promote sustainable improvements in the prescribing practice. Competing demands and patient influences remain important challenges, which future studies must address to facilitate the reduction of PIPs in primary care.

## Supplementary Information

**Additional file 1.** Standards for Reporting Qualitative Research (SRQR).

## Data Availability

No datasets are available from this study owing to the consents given by participants, which limits data to the research team only.

## Abbreviations

PIP: Potentially inappropriate prescribing
QI: Quality improvement
NPT: Normalisation Process Theory
GP: General practitioner
OGP: Organisation of General Practitioners
DDD: Defined daily dose
PPI: Proton-pump inhibitor
EMR: Electronic medical record
