# Supplementary material for: Exploring implementation processes in general practice in a feedback intervention aiming to reduce potentially inappropriate prescribing: a qualitative study among general practitioners
Source: Implement Sci Commun. 2021 Jan 7;2:4. doi: 10.1186/s43058-020-00106-5 (PMC7792001; doi:10.1186/s43058-020-00106-5)
Supplement: Supplementary file 1 — Additional file 1. Standards for Reporting Qualitative Research (SRQR). [file 43058_2020_106_MOESM1_ESM.docx]

| Standards for Reporting Qualitative Research (SRQR) | | |
| --- | --- | --- |
| No. | Topic | Page (section) in manuscript |
|  | **Title and abstract** |  |
| S1 | Title | P. 1 (Title page) |
| S2 | Abstract | P. 2-3 (Title page) |
|  | **Introduction** |  |
| S3 | Problem formulation | P. 4-5 (Introduction) |
| S4 | Purpose or research question | P. 6 (Introduction) |
|  | **Methods** |  |
| S5 | Qualitative approach and research paradigm | P. 5-6 (Introduction + Design and setting) |
| S6 | Researcher characteristics and reflexivity | P. 25 (Discussion) + p. 27 (Author´s information) |
| S7 | Context | P. 7 (Implementation context) |
| S8 | Sampling strategy | P. 7-8 (Sampling strategy) |
| S9 | Ethical issues pertaining to human subjects | P. 26 (Ethics approval and consent to participate) |
| S10 | Data collection methods | P. 8 (Data collection) |
| S11 | Data collection instruments and technologies | P. 8 (Data collection) |
| S12 | Units of study | P. 7-8 (Sampling strategy) + p.9 (Results) |
| S13 | Data processing | P. 8 (Data collection) + p. 26 (Ethics approval and consent to participate) |
| S14 | Data analysis | P. 8 (Data analysis) |
| S15 | Techniques to enhance trustworthiness | P. 8 (Data analysis) + 25 (Discussion) |
|  | **Results/findings** |  |
| S16 | Synthesis and interpretation | P. 9-19 (Results) |
| S17 | Links to empirical data | P. 9-19 (Results) |
|  | **Discussion** |  |
| S18 | Integration with prior work, implications,  transferability, and contribution(s) to the field | P. 19-24 (Discussion) |
| S19 | Limitations | P. 24-25 (Discussion) |
|  | **Other** |  |
| S20 | Conflicts of interest | P. 26 (Competing interests) |
| S21 | Funding | P. 26 (Funding) |
|  |  |  |
